# Alzheimer’s Disease Phenotype or Inflammatory Insult Does Not Alter Function of L-Type Amino Acid Transporter 1 in Mouse Blood-Brain Barrier and Primary Astrocytes

**DOI:** 10.1007/s11095-018-2546-7

**Published:** 2018-11-28

**Authors:** Mikko Gynther, Elena Puris, Soile Peltokangas, Seppo Auriola, Katja M. Kanninen, Jari Koistinaho, Kristiina M. Huttunen, Marika Ruponen, Kati-Sisko Vellonen

**Affiliations:** 1 School of Pharmacy, University of Eastern Finland, P.O. Box 1627, FI-70211 Kuopio, Finland; 2 A.I. Virtanen Institute for Molecular Sciences, University of Eastern Finland, FI-70211 Kuopio, Finland

**Keywords:** alzheimer’s disease, blood-brain barrier, CNS drug delivery, L-type amino acid transporter

## Abstract

**Purpose:**

The study aim was to evaluate the effect of Alzheimer’s disease (AD) and inflammatory insult on the function of L-type amino acid transporter 1 (Lat1) at the mouse blood-brain barrier (BBB) as well as Lat1 function and expression in mouse primary astrocytes.

**Methods:**

The Lat1 function and expression was determined in wildtype astrocytes with and without lipopolysaccharide (LPS)-induced inflammation and in LPS treated AD *APP/PS1* transgenic astrocytes. The function of Lat1 at the BBB was evaluated in wildtype mice with and without LPS-induced neuroinflammation and *APP/PS1* transgenic mice by *in situ* brain perfusion.

**Results:**

There were 2.1 and 1.6 -fold decreases in Lat1 mRNA and protein expression in LPS-treated wildtype astrocytes compared to vehicle-treated astrocytes. In contrast, Lat1 mRNA and protein expression were increased by 1.7 and 1.2 -fold (not statistically significant) in the transgenic cells. A similar trend was observed in the cell uptake of [^14^C]-L-leucine. There were no statistically significant differences in [^14^C]-L-leucine BBB permeation between the groups.

**Conclusions:**

The results showed that neither LPS-induced inflammation or the presence of *APP/PS1* mutations alters Lat1 function at the mouse BBB as well as Lat1 protein expression and function in mouse primary astrocytes.

## Introduction

One of the major challenges in the development of new drugs for central nervous system (CNS) disorders such as Alzheimer’s disease (AD) is the delivery of the drug molecules into the site of action. (1) The blood-brain barrier (BBB) protects the brain efficiently by controlling the movement of solutes from blood to brain by the means of enzymes, transporters and tight junctions between the endothelial cells. In addition, transporter proteins expressed in brain parenchyma have a deciding role in the intra-brain distribution of drugs. (2,3) This complicates the CNS drug delivery and can hinder the development of efficient drug therapies. (4) There is a great need for drug delivery technologies across the BBB and into the target cells. One approach to overcome the BBB is to design drugs or prodrugs capable of utilizing influx transporters highly expressed at the BBB. (4) The same transporter can be used to reach the target cells in the brain parenchyma. The prerequisite for transporter mediated drug delivery approach is, however, profound knowledge of transporter expression and function in the diseased brain (5).

L-type amino acid transporter 1 (Lat1) is a heterodimeric transporter protein capable of transporting neutral, large and branched amino acids, such as leucine and tryptophan, across the cell membrane. (6) In addition, Lat1 has been shown to transport many CNS drugs, such as L-DOPA and gabapentin across the BBB into the brain. (7,8) Moreover, there are numerous reports of Lat1 being utilized for the delivery of prodrugs of CNS acting agents into the mouse or rat brain and into the intracellular compartment of brain parenchyma. (9–12) Lat1 is expressed on both luminal and abluminal brain capillary membranes and the membrane of cells within the brain parenchyma. (13,14) This enables Lat1-utilizing compounds to cross not only the BBB but also to penetrate cell membranes in the brain. However, there are concerns whether influx transporters, in particular Lat1, retain sufficient expression and function in the brain and BBB in neuropathological conditions. For example, glucose transporter type 1 (GLUT1/Glut1) is downregulated in brain parenchymal cells and the BBB of AD patients and AD mouse models. (15–17) In Parkinson’s disease, the usefulness of L-DOPA, an Lat1 utilizing prodrug of dopamine, proves that the Lat1 transporter function remains efficient despite the disease. (7) However, the expression and function of Lat1 transporter in the BBB and brain parenchyma suffering from AD and neuroinflammation has not been explored in the literature extensively.

In order to investigate the effect of AD on Lat1 expression, an *APP/PS1* transgenic mouse model with mutations in genes encoding amyloid precursor protein (*APP*) and presenilin (*PSEN1*) was utilized. (18) The effects of the mutations on Lat1 expression and function at the BBB and astrocytes are sparse. Vellonen *et al*. (19) recently reported the mRNA expression of Lat1 in *APP/SPS1* mouse brain capillaries and no significant differences were found compared to wildtype control mice. However, neither Lat1 protein expression nor function were investigated in that study. There exists a recent report of reduced expression of Lat1 in CNS disease models of neuroinflammation. (20) The results demonstrated that LPS-induced inflammation rapidly decreased Lat1 mRNA expression at the BBB of male mice and rats, while changes in Lat1 protein level followed a slower kinetics. Importantly, the effect of LPS-induced neuroinflammation on the function of Lat1 at the mouse BBB has not been studied.

Astrocytes control the brain ion homeostasis, regulate pH and blood flow in brain. In addition, astrocytes have important neuro-supportive role as they contribute to synaptogenesis, synaptic transmission, synaptic structural integrity. (21) Importantly, astrocytes play a significant role in the development of AD. As several target proteins for potential AD drugs reside inside the astrocytes, it makes the transporter expression in these cells of great interest in terms of drug delivery. (21) Moreover, as Lat1 has a significant role in the intra-brain distribution of its substrates, (9,22,23) it is important to elucidate whether the Lat1 expression and function are altered in astrocytes due to existing inflammation or AD transgene expression. The decreased transporter function at the BBB and in the target cells due to low protein expression would mitigate the utilization of Lat1 for brain drug delivery in neurodegenerative diseases such as AD.

The aim of the present study was to investigate whether LPS-induced acute inflammation or the presence of AD-causing mutations in *APP* and *PS1* genes affect the function of Lat1 at the mouse BBB, as well as the Lat1 mRNA and protein expression and function in primary mouse astrocytes. The Lat1 function at the BBB of wildtype, LPS-treated and AD transgenic mice was investigated by measuring the BBB permeation rate via unidirectional transfer constant (K_in_) of Lat1 substrate [^14^C]-L-leucine using the *in situ* mouse brain perfusion technique. The Lat1 function in wildtype astrocytes as well as wildtype and AD transgenic astrocytes treated with LPS was determined and [^14^C]-L-leucine K_m_ and V_max_ values calculated. In addition, the Lat11 protein expression in the astrocytes was quantified by liquid chromatography-mass spectrometry (LC-MS/MS)-based proteomics.

## Materials and Methods

### Animals

Mice carrying human *APP* (K595 N and M596 L) and *PSEN1dE9* mutations maintained in C57BL/6 J background were used as a mouse model of AD (18) (Jackson Laboratories, Bar Harbor, ME, USA). These mice are referred to as *APP/PS1* mice. Aβ plaques appear in this model from approximately the age of four-five months and animals were used for the experiments between the ages of 7 to 8 months. Age-matching C57BL/6 J strain wildtype mice were used as controls. LPS treated mice were of the C57BL/6JOIaHsd strain and were 12 weeks old at the time of the experiments. The animals were kept in controlled environment with 12 h light/dark cycle and food and water freely available. The animal studies were performed according to the Council of Europe (Directive 86/609) and Principles of laboratory Animal Care. Studies were approved by the local committee of animal experiments (ESAVI/3347/04.10.07/2015).

### ***In Situ*** Mouse Brain Perfusion

Mice were anesthetized with intraperitoneal (i.p.) injections of ketamine (120 mg/kg) and xylazine (8 mg/kg), and their right carotid artery system was exposed. The right external carotid artery was ligated, and the right common carotid artery was cannulated with a catheter filled with 100 IU/mL heparin. The perfusions were performed at 37°C with a flow rate of 2.5 mL/ min for 30 s followed by the washing of the capillaries for 2 s with 4°C drug-free perfusion buffer. The function of Lat1 at the BBB was evaluated by perfusing 0.157 μM [^14^C]-L-leucine (PerkinElmer, Waltham, MA, USA) as a known Lat1 probe substrate. The Lat1 function was assessed in *APP/PS1* transgenic mice, wildtype control mice and wildtype mice injected with 250 μg/kg (i.p.) of LPS once a day for three days followed by the brain perfusion performed 24 h after the final injection. In addition, a group of wildtype mice without LPS treatment were perfused with a combination of [^14^C]-L-leucine and 100 μM of selective Lat1 inhibitor (S)-2-Amino-3-(3-((2,4-dicyano-3-(4-(2-(methylamino)-2oxoethoxy)phenyl)benzo (3,5) imidazo[1,2-a]pyridin-1-yl)-carbamoyl)phenyl)propanoic acid (KMH-233). (24) After the brain perfusion the hippocampus and cerebral cortex of the right cerebral hemisphere were collected and the K_in_ of [^14^C]-L-leucine was determined (10).

### Astrocyte Cultures

Primary astrocytes were isolated from 2-day-old wildtype and *APP/PS1* mice and cultured as described earlier. (25) Shortly, cortices and hippocampi were isolated and the tissue was suspended into DMEM medium (Fisher Scientific GmbH, Schwerte, Germany) containing 10% heat-inactivated fetal bovine serum (Fisher Scientific GmbH, Schwerte, Germany) and 100 U/mL penicillin streptomycin (Fisher Scientific GmbH, Schwerte, Germany). The suspension was triturated ten times and thereafter centrifuged 1500 rpm for 5 min at room temperature. Trypsin-EDTA (Fisher Scientific GmbH, Schwerte, Germany) of 0.25% was added and the suspension was incubated for 30 min at 37°C. Fresh culture medium was added and the suspension was centrifugated 1500 rpm for 5 min. The cells were plated onto the poly-L-lysine (Sigma-Aldrich, Darmstadt, Germany) coated flasks in culture medium. To remove microglia, the cultures were shaken at 200 rpm for 2 h before cells were used for experiments.

For uptake experiments the astrocytes (passage 7–16) were seeded on 24-well plates (10^4^ cells/well) three days before experiments. The cells were cultured at 37 °C, 5% CO_2_ in culture medium (Gibco, DMEM/F12, Fisher Scientific GmbH, Schwerte, Germany) containing 10% heat-inactivated fetal bovine serum and 100 U/mL penicillin streptomycin). When effect of inflammation was studied, the cells were exposed to 100 ng/mL LPS (Sigma-Aldrich, Darmstadt, Germany) for 24 h before the uptake experiment.

### Uptake Experiment

Astrocytes were preincubated with washing buffer (Gibco HBSS, Schwerte, Germany), 25 mM Hepes (Sigma-Aldrich, Darmstadt, Germany), pH 7.4 for 10 min at 37°C. Washing buffer was replaced by the reaction buffer (HBSS, 25 mM Hepes, pH 7.4 containing 0.25 μCi/mL [^14^C]-L-leucine (PerkinElmer, Inc., Waltham, MA, USA) and incubated for 2.5 min at 37°C. Thereafter, the cells were washed three times with ice-cold buffer and lysed using 0.1 N NaOH for 60 min. The cell lysate was mixed with Ultima Gold scintillation cocktail (PerkinElmer, Inc., Waltham, MA, USA) and radioactivity was measured with MicroBeta liquid scintillation counter (Wallac Oy, Finland). The protein concentration of the samples was determined using Bio-Rad protein assay (Bio-Rad Laboratories, Germany). Experiments were repeated 3–4 times with 3 parallel samples.

Inhibition of [^14^C]-L-leucine uptake was studied by adding 2 mM tryptophan to the reaction buffer. In the experiments where concentration dependent uptake of L-leucine was studied, desired concentration of non-labeled L-leucine (1–400 μM) was added to the reaction buffer. The lowest concentration contained 0.05 μCi/mL of [^14^C]-L-leucine.

### Quantitative RT-PCR

The mRNA expression of Lat1 (Slc7a5 gene) was investigated in astrocytes isolated from wildtype and *APP/PS1* transgenic mice. RNA was extracted with RNeasy Micro Kit (Qiagen, Germany) according to manufacturer’s protocol. DNase treatment was done with DNA-free kit (Ambion, USA) and RNA concentration was measured with NanoVue (GE Healhcare, UK). RNA of 200 ng was transcribed into cDNA by using M-Mulv reverse transcriptase and random primers (Fermentas, USA). cDNA corresponding to 4 ng of RNA was amplified using SYBR Green chemistry, primers designed for Slc7a5 (forward CTCCTTGCCCATTGTCACAC, reverse GGACATGACACCCAAGTGGTAG) and ABI Prism 7500 (Applied Biosystems, UK). Melting curve analysis was performed after amplification cycles in order to check the specificity of amplification. Gapdh Endogenous Control (Applied Biosystems, UK) was used for normalization of Slc7a5 expression. Normalized expression was calculated with the QGene application using amplification efficiency determined for the Slc7a5 primer pair (26).

### Transporter Protein Quantitation from Astrocyte Crude Membrane Fractions

ProteoExtract Subcellular Proteome Extraction Kit (Merck KGaA, Darmstadt, Germany) was used for the isolation of crude membrane fraction from the cell pellets of astrocytes following the manufacturer’s instructions. The protein concentrations in the crude membrane fractions were determined as mean of three samples by Bio-Rad Protein Assay (EnVision, PerkinElmer, Inc., Waltham, MA, USA), and aliquots containing 50 μg of total protein were taken for further sample preparation.

#### Quantitative Protein Expression of Lat1 and Glut1 with Multiple Reaction Monitoring (MRM) by LC − MS/MS

The protein expression of Lat1 and Glut1 in mouse wildtype and AD transgenic astrocytes was quantified by means of multiplexed MRM analysis according to the protocol described by Uchida *et al*., 2013. (27) Briefly, the aliquots of crude membrane cell fractions containing 50 μg of protein were solubilized in 500 mM Tris–HCl (pH 8.5), 7 M guanidine hydrochloride and 10 mM EDTA. Consequently, the proteins were *S*-carbamoylmethylated with dithiothreitol followed by iodoacetamide treatment. After alkylation, the proteins were precipitated with methanol and chloroform. The dissolution of precipitates was performed by addition of 6 M urea dissolved in 100 mM Tris–HCl (pH 8.5) followed by 5-fold dilution with 0.1 M Tris–HCl (pH 8.5) spiked with internal standard peptide (JPT Peptide Technologies GmbH, Berlin, Germany). After treatment with Protease-Max surfactant (Promega, Madison, WI, USA) and digestion with lysyl endopeptidase (Lys-C: Wako Pure Chemical Industries, Osaka, Japan) at 30°C for 3 h, tosylphenylalanyl chloromethyl ketone-treated trypsin (Promega, Madison, WI, USA) was added into the samples (enzyme/substrate ratio of 1:100). For tryptic digestion samples were incubated at 37°C for 16 h. Before LC − MS/MS analysis, the digests were acidified with 20% (*v*/v) formic acid in water and centrifuged at 15000×g for 5 min at 4°C.

LC − MS/MS analysis was conducted using an Agilent 1290 Infinity LC (Agilent Technologies, Waldbronn, Germany) instrumentation coupled to an Agilent 6495 Triple Quadrupole Mass Spectrometer equipped with an electrospray ionization (ESI) source (Agilent Technologies, Palo Alto, CA, USA). The peptides were separated and eluted with the HPLC method described by Gynther *et al*. (28) The conditions for peptide detection were used as follows: ESI positive ion mode, the source temperature was maintained at 210°C, drying gas (nitrogen) flow rate was 16 L/min, nebulizer pressure was 45 psi, the MS capillary voltage was 3 kV. Dwell time was 20 ms. MRM mode was applied. For the quantitation of the target protein, one unique peptide was chosen according to the in silico peptide selection criteria published by Uchida *et al*. 2013 (27) with exception to methionine residue included to the structure of peptide. Three MRM transitions for Lat1 specific peptide corresponding to high intensity fragment ions were selected for a stable isotope-labeled peptide and the unlabeled natural peptide (Table I). Data were acquired using the software Agilent MassHunter Workstation Acquisition (Agilent Technologies, Data Acquisition for Triple Quad., version B.03.01). The data processing and analysis were performed with Skyline v 4.1 software.

**Table I:** Standard (St) and Internal Standard (Is) Probe Peptides and MRM Transitions for the LC-MS/MS Analysis of Lat1 and Glut1

Protein	St/Is	Probe peptide sequence	Retention time (min)	MRM transitions (m/z)			
				Q1	Q3–1	Q3–2	Q3–3
Lat1	St	DMGQGDASNLQQK	9.6	696.3	960.4	788.4	403.2
	Is	DMGQGDASNLQQK*	9.6	700.3	968.4	796.4	411.2
Glut1	St	TFDEIASGFR	21.3	571.8	894.4	537.3	
	Is	TFDEIASGFR*	21.3	576.8	904.4	547.3	

* denotes ^13^C labeled arginine and lysine

### Data Analysis

All statistical analyses were performed using GraphPad Prism v. 5.03 software (GraphPad Software, San Diego, CA, USA). The data is presented as mean ± SD.

## Results and Discussion

### Lat1 mRNA and Protein Expression in Mouse Primary Astrocytes

Astrocytes are important targets for therapeutic compounds against AD as well as neuroinflammation and as Lat1 expression and function is important for the intra-brain distribution and particularly uptake of substrate drugs into their target cells, we investigated the effect of LPS induced inflammation and AD-causing mutations in *APP* and *PS1* genes on Lat1 mRNA and protein expression in mouse primary astrocytes. Given that inflammatory reaction and reactive astrogliosis have been recognized as integral parts of AD pathology, the astrocytes were treated with LPS to activate them. We have previously shown that the LPS treatment of primary astrocytes increases the secretion of IL6 and MCP-1. (29) The elevated levels of these cytokines have also been measured from AD patients. (30,31) The effect of LPS and AD transgenes on Lat1 mRNA expression was investigated by qRT PCR and protein expression was investigated by quantitative LC-MS/MS based proteomics.

**Fig. 1:**
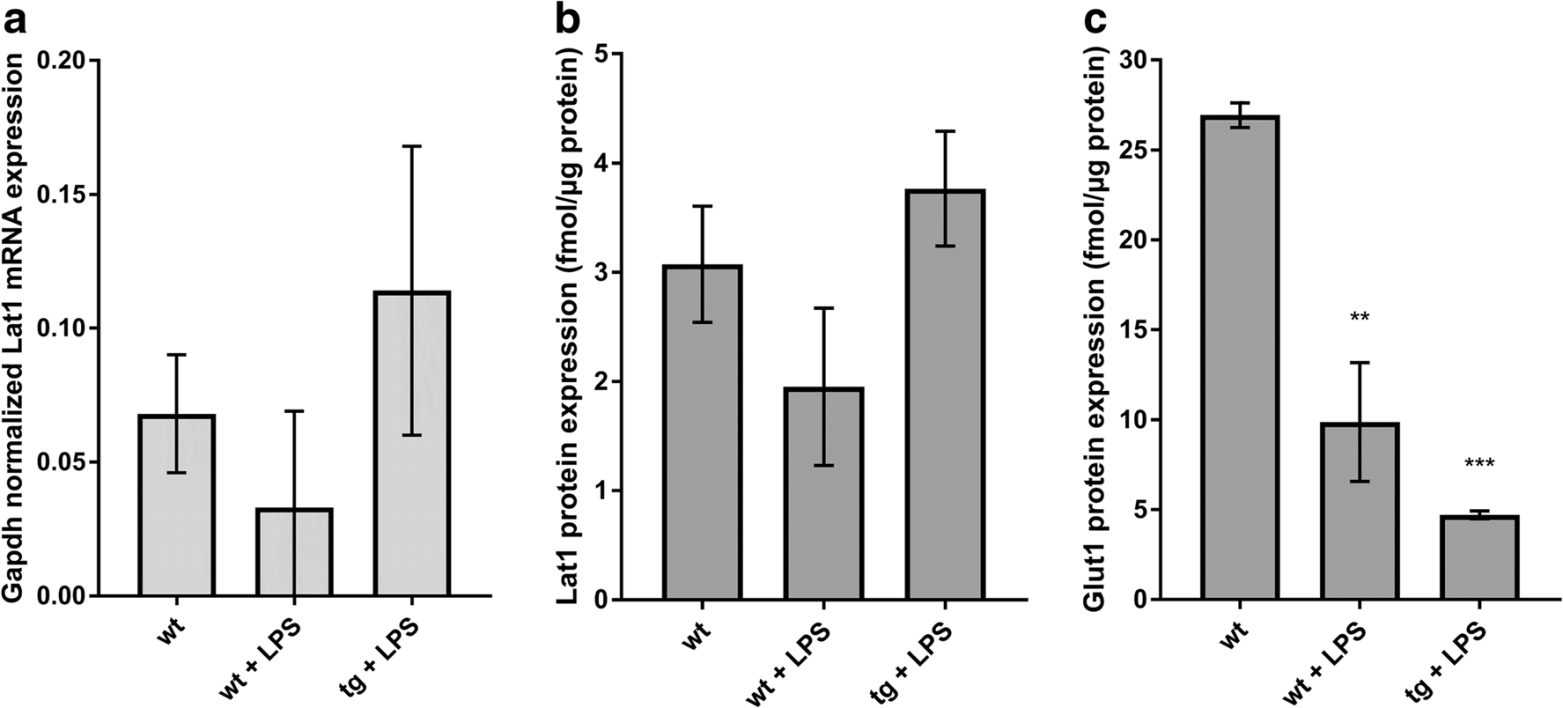
The Lat1 mRNA expression (**a**) and protein level (**b**) in wildtype astrocytes with and without LPS treatment and AD transgenic mouse astrocytes with LPS treatment. Glut1 protein level (**c**) in wildtype astrocytes with and without LPS treatment and AD transgenic mouse astrocytes with LPS treatment. The data is presented as mean ± SD, *n* = 3. The statistical difference between the groups was investigated using one-way ANOVA, followed by Dunnett’s test, **P < 0.01, ***P < 0.001.

The expression of Lat1 mRNA and protein was seen in both wildtype and AD transgenic astrocytes with and without LPS-induced inflammation. The Lat1 mRNA levels were 0.068 ± 0.022, 0.033 ± 0.036 and 0.114 ± 0.054 (Gapdh normalized expression, mean ± SD) in vehicle-treated wildtype, LPS-treated wildtype and LPS-treated AD astrocytes, respectively (Fig. 1a). The protein expression in vehicle-treated wildtype, LPS-treated wildtype and LPS-treated transgenic cells were 3.07 ± 0.92, 1.95 ± 1.25 and 3.77 ± 0.91 fmol/μg protein (mean ± SD), respectively (Fig. 1b). Interestingly, there was a trend towards a decrease of both Lat1 mRNA (2.1-fold) and protein (1.6-fold) in the LPS-treated wildtype astrocytes when compared to the vehicle-treated wildtype cells. In contrast, the mRNA (1.7-fold) and protein (1.2-fold) expressions were higher in the transgenic astrocytes compared to the wildtype cells, although none of the changes were statistically significant.

In order to confirm the phenotype of the AD transgenic astrocytes, we determined the protein level of Glut1 in the astrocyte samples, as it is known to be downregulated in the astrocytes of AD brain. (15–17) Unlike Lat1 protein expression, the Glut1 protein expression was significantly decreased in both wildtype cells treated with LPS and in the AD transgenic cells (Fig. 1c). The Glut1 protein expression was 26.93 ± 1.16, 9.87 ± 5.73 and 4.70 ± 0.38 fmol/μg protein (mean ± SD), for vehicle-treated wildtype cells, wildtype cells treated with LPS and transgenic astrocytes treated with LPS, respectively. This is in accordance with literature reports of reduced GLUT1 expression in the AD brain. (15–17) However, instead of direct effects, the *APP* and *PS1* mutations should have indirect consequences on astrocyte biology since the mutations are under the control of a prion promoter, which is considered to be primarily neuronal. Although Glut1 expression was significantly downregulated in the *APP* and *PS1* transgenic astrocytes compared to the wildtype cells, indicating AD phenotype, caution has to be used in interpreting these results before confirming them in astrocytes derived from adult mice with *APP* and *PS1* mutations.

### Lat1 Function in Mouse Primary Astrocytes

**Fig. 2:**
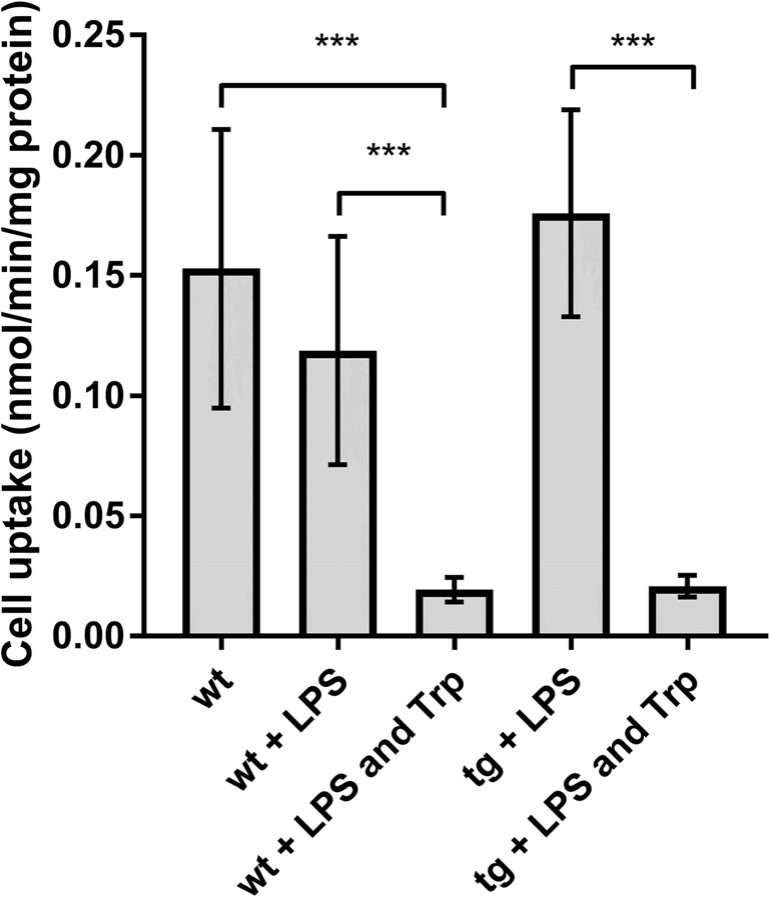
The cell uptake of 0.157 μM [^14^C]-L-leucine in wildtype and AD astrocytes with and without LPS treatment and presence of 2 mM L-tryptophan. The data is presented as mean ± SD, n = 3. The statistical difference between the groups was determined using one-way ANOVA, followed by Tukey’s test, ***P < 0.001.

The function of Lat1 in AD transgenic and wildtype astrocytes was investigated by measuring the cell uptake of Lat1 substrate [^14^C]-L-leucine (Fig. 2). The Lat1-mediated cell uptake of [^14^C]-L-leucine was confirmed by inhibiting the cell uptake by competing Lat1 substrate L-tryptophan. In both wildtype and AD astrocytes the presence of 2 mM L-tryptophan reduced the cell uptake of [^14^C]-L-leucine significantly by approximately 10 times. The cell uptake of [^14^C]-L-leucine was 0.153 ± 0.058, 0.019 ± 0.005, 0.227 ± 0.043 and 0.021 ± 0.004 nmol/mg protein (mean ± SD) for wildtype cells, wildtype cells with L-tryptophan, transgenic cells and transgenic cells with L-tryptophan, respectively. In wildtype astrocytes LPS reduced the [^14^C]-L-leucine cell uptake by 1.3-fold (0.119 ± 0.048 nmol/mg protein) compared to the non-treated wildtype cells, but the change was not statistically significant. There was no statistically significant difference in leucine uptake between vehicle-treated wildtype cells, wildtype cells treated with LPS and transgenic cells treated with LPS, which is in line with the Lat1 protein expression results.

**Fig. 3:**
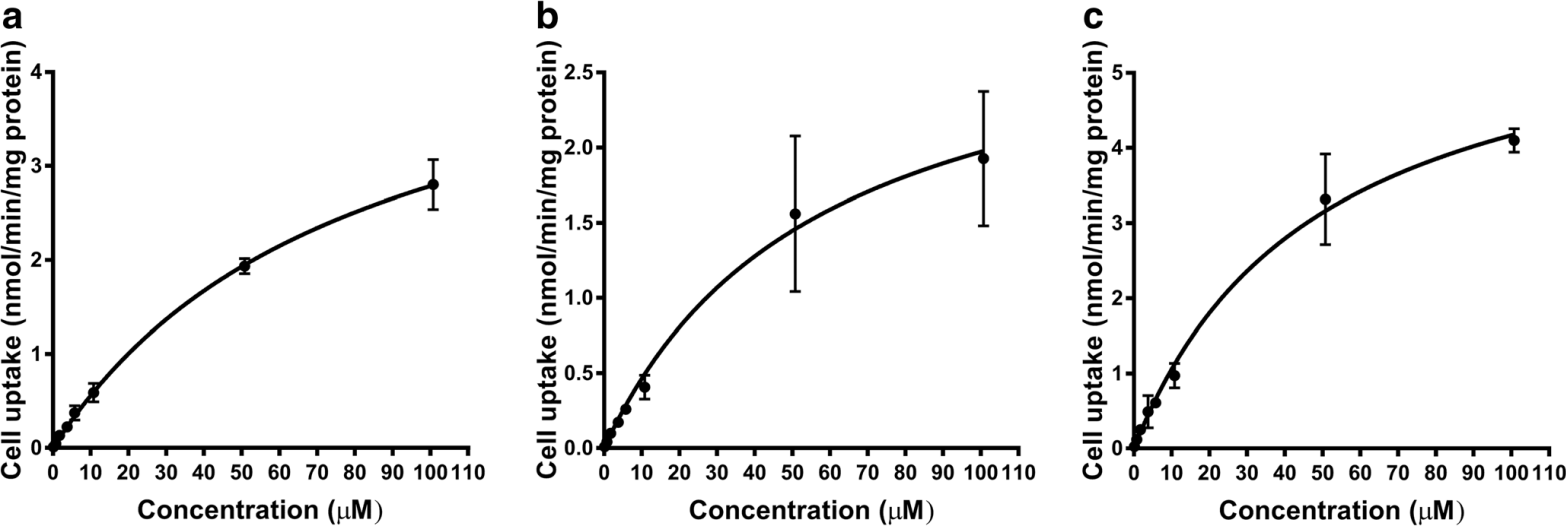
The concentration dependent cell uptake of for [^14^C]-L-leucine in LPS treated AD astrocytes (**a**), wildtype astrocytes without LPS (**b**) and wildtype astrocytes with LPS (**c**). The data is presented as mean ± SD, n = 3–4.

The concentration-dependent uptake of [^14^C]-L-leucine revealed saturable kinetics (Fig. 3). Neither LPS-induced inflammation nor the presence of AD-causing transgenes altered significantly the function of Lat1 in mouse primary astrocytes compared to the non-treated wildtype cells (Table II). There was a trend that the uptake capacity via Lat1 (V_max_) was decreased in the LPS treated wildtype cells (1.5-fold decrease) and increased (1.3-fold) in the AD transgenic astrocytes compared to wildtype cells. This is in accordance with the changed Lat1 expression and cell uptake results.

**Table II:** The Michaelis-Menten Kinetic Parameters for [^14^C]-L-leucine Cell Uptake. The Data is Presented as Mean ± SD, n = 3–4. Statistical Significances of Differences Between the Groups were Tested by One-Way ANOVA Followed by Tukey’s Test

	wildtype	wildtype + LPS	transgenic + LPS
V_max_ (nmol/min/mg)	4.9 ± 1.3	3.2 ± 0.9	6.3 ± 0.3
K_m_ (μM)	73.3 ± 17.7	59.7 ± 18.4	51.6 ± 11.9

### Lat1 Function at the BBB of LPS Treated and AD Transgenic Mice

**Fig. 4:**
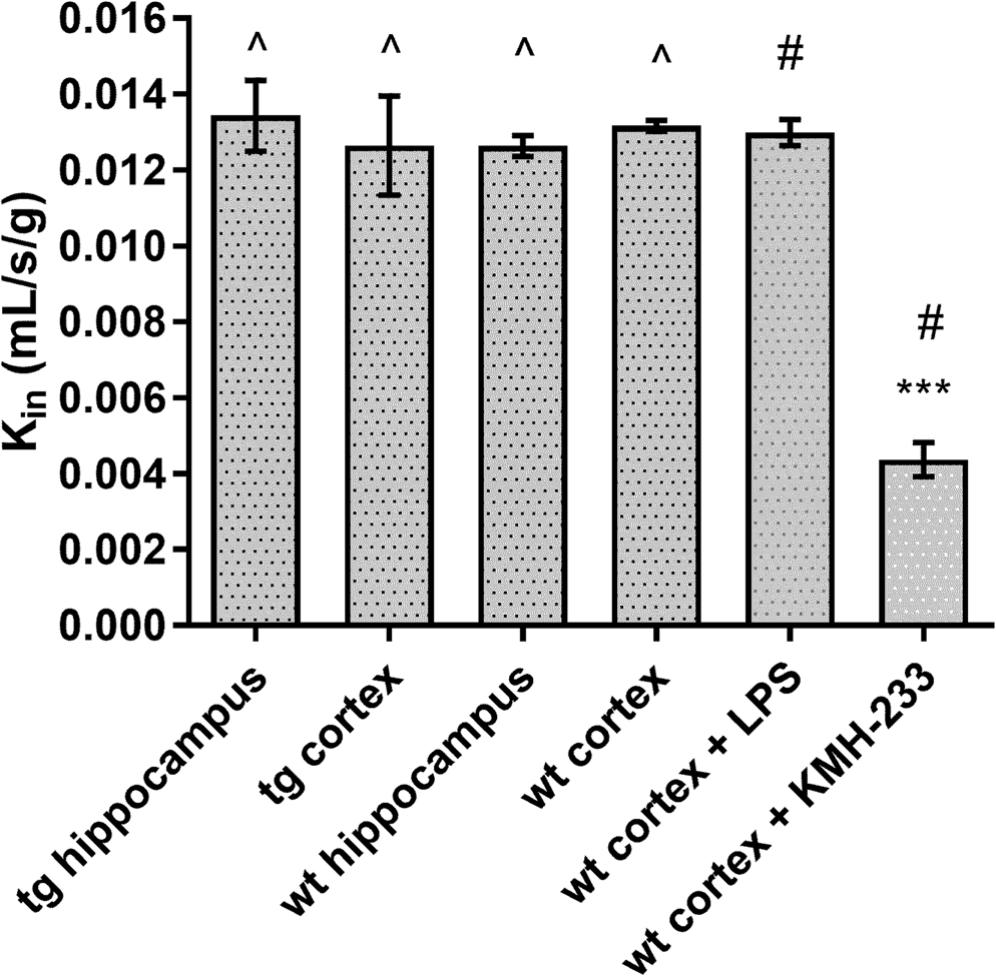
The K_in_ values of [^14^C]-L-leucine after mouse brain perfusion. The data is presented as mean ± SD (n = 3–5). The statistical difference between the groups was determined using one-way ANOVA, followed by Tukey’s test, ***P < 0.001. ^ denotes that the mice used in the experiments were 7–8 months old, whereas # denotes that the mice were 3 months old.

We investigated the effect of LPS-induced acute inflammation on the Lat11 function in mouse cerebral cortex BBB. The mice were injected i.p. with 250 μg/kg of LPS for three consecutive days, a dose and regimen which has been previously shown to induce amylodogenesis and neuroinflammation in mice. (32) However, the dose is not high enough to compromise the tight junction integrity at the BBB and cause paracellular brain permeation of hydrophilic compounds such as amino acids. Banks *et al*. (33) reported that more than 10-fold higher (3 mg/kg) dose is required for BBB disruption. The function of Lat1 at the BBB was investigated using *in situ* brain perfusion in mice performed 24 h after the last LPS injection (Fig. 4). The results showed that there were no changes in the BBB permeation rate measured by K_in_ values of [^14^C]-L-leucine due to LPS-induced neuroinflammation compared to the non-treated control mice. Whereas, co-perfusion of [^14^C]-L-leucine with 100 μM of selective Lat1 inhibitor KMH-233 reduced the [^14^C]-L-leucine uptake significantly. In addition, we investigated the effect of AD-causing transgenes on Lat1 function in mouse cerebral cortex and hippocampus using the same method. Interestingly, no changes were observed in the Lat1 function compared to the control or LPS treated mice. The lack of LPS effect is in contradiction with the BBB expression results reported earlier. (20) In the study by Wittmann *et al*., there was significant and time dependent reduction of both Lat1 mRNA and protein levels at the BBB of LPS treated mice. However, whether the reduction of Lat1 expression was enough to cause a significant effect in Lat1 function at the BBB was not investigated. In the present study the possible changes in Lat1 protein expression due to LPS-induced inflammation or presence of AD-causing mutations in *APP* and *PS1* genes are not significant enough to affect the function of the transporter at the BBB. Moreover, it has to be noted that the dose and regimen of LPS treatment was different in the present study and the study by Wittmann *et al*. (20) where the mice were injected once with 2.5 mg/kg. Therefore, there might be differences in the changes of Lat1 expression at the mouse BBB between these two studies.

## Conclusions

The results of the present study showed that there were no significant changes in the Lat1 function at the BBB of LPS-induced inflammation mouse model and AD transgenic mice compared to non-treated wildtype mice. In addition, there were no changes in the mRNA or protein levels in LPS-treated wildtype and AD transgenic primary mouse astrocytes compared to vehicle-treated wildtype astrocytes. Moreover, this is reflected in the unaltered Lat1 function in the LPS-treated and transgenic cells. All together, these data support the view that Lat1 can be utilized for the delivery of potential AD drugs across the BBB and astrocyte cell membrane.

## Abbreviations

Aβ: β-amyloid
AD: Alzheimer’s disease
BBB: Blood-brain barrier
CNS: Central nervous system
Lat1: L-type amino acid transporter 1
LC-MS/MS: Liquid chromatography mass spectrometry
LPS: Lipopolysaccharide
MRM: Multiple reaction monitoring
